# De Novo Transcriptome Assembly, Gene Annotations, and Characterization of Functional Profiling Reveal Key Genes for Lead Alleviation in the Pb Hyperaccumulator Greek Mustard (*Hirschfeldia incana* L.)

**DOI:** 10.3390/cimb44100318

**Published:** 2022-10-04

**Authors:** Said El Hasnaoui, Mouna Fahr, Mohamed Zouine, Abdelaziz Smouni

**Affiliations:** 1 Laboratoire de Biotechnologie et Physiologie Végétales, Centre de Biotechnologie Végétale et Microbienne Biodiversité et Environnement, Faculté des Sciences, Université Mohammed V de Rabat, Rabat 10000, Morocco; 2 Laboratoire Mixte International Activité Minière Responsable “LMI-AMIR”, IRD/UM5/INAU, Rabat 10000, Morocco; 3 Laboratoire de Recherche en Sciences Végétales, Université de Toulouse, CNRS, UPS, Toulouse INP, 31320 Auzeville-Tolosane, France

**Keywords:** *Hirschfeldia incana* L., Pb stress, RNA-seq, de novo assembly, transcriptome, jasmonic acid

## Abstract

Lead (Pb) contamination is a widespread environmental problem due to its toxicity to living organisms. *Hirschfeldia incana* L., a member of the Brassicaceae family, commonly found in the Mediterranean regions, is characterized by its ability to tolerate and accumulate Pb in soils and hydroponic cultures. This plant has been reported as an excellent model to assess the response of plants to Pb. However, the lack of genomic data for *H. incana* hinders research at the molecular level. In the present study, we carried out RNA deep transcriptome sequencing (RNA-seq) of *H. incana* under two conditions, control without Pb(NO_3_)_2_ and treatment with 100 µM of Pb(NO_3_)_2_ for 15 days. A total of 797.83 million reads were generated using Illumina sequencing technology. We assembled 77,491 transcript sequences with an average length of 959 bp and N50 of 1330 bp. Sequence similarity analyses and annotation of these transcripts were performed against the *Arabidopsis thaliana* nr protein database, Gene Ontology (GO), and KEGG databases. As a result, 13,046 GO terms and 138 KEGG maps were created. Under Pb stress, 577 and 270 genes were differentially expressed in roots and aboveground parts, respectively. Detailed elucidation of regulation of metal transporters, transcription factors (TFs), and plant hormone genes described the role of actors that allow the plant to fine-tune Pb stress responses. Our study revealed that several genes related to jasmonic acid biosynthesis and alpha-linoleic acid were upregulated, suggesting these components’ implication in *Hirschfeldia incana* L responses to Pb stress. This study provides data for further genomic analyses of the biological and molecular mechanisms leading to Pb tolerance and accumulation in *Hirschfeldia incana* L.

## 1. Introduction

Lead (Pb) is known as one of the prominent heavy metals that occurs in the environment both from natural sources and mainly from anthropogenic activities [1]. Pb is known to be toxic to plants, animals, and microorganisms [1]. As one of the non-essential elements, Pb has no biological function and is toxic to living organisms even at low concentrations [2]. In plants, Pb causes various morphological, physiological, and biochemical effects [3]. However, several plant species have developed various tolerance strategies that are used to mitigate Pb toxicity [2]. These plant species can reduce the levels of Pb toxicity by the induction of both enzymatic and non-enzymatic pathways [4]. In *Brassica juncea*, it was reported that Pb toxicity increases SOD (superoxide dismutase) and APX (ascorbate peroxidase) levels with a decrease in CAT (Catalase) activity [5]. Hyper-tolerant species such as *Festuca ovina, Silene vulgaris, Noccaea caerulescens*, and *Peganum harmala* L. absorb and accumulate high levels of Pb in their root tissues and restrict Pb translocation to the aboveground parts [6,7]. Interestingly, plants with the capacity to hyper-accumulate Pb can accumulate concentrations higher than 1000 mg Kg^−1^ of Pb in their aerial parts without showing toxicity symptoms [8]. *Hirschfeldia incana* L. commonly known as shortpod mustard is a perennial plant belonging to the Brassicaceae family currently found in the Mediterranean area and known for its capacity to grow and survive on highly heavy-metal-contaminated soils [9,10]. This plant has been identified and characterized as a Pb hyper-accumulator and a good model to evaluate the response of plants to Pb at physiological and molecular levels [8,10,11]. In addition, *H. incana* accumulates higher than 3 percent dry weight of Pb in its aerial parts when cultivated in a hydroponic medium containing 100 μM of Pb(NO_3_)_2_ [8,10]. Several plants have developed and formed specific physiological and molecular mechanisms to cope with Pb stress successfully. To date, many scientific reports have been published about the effects of Pb on plants and their tolerance mechanisms [2], but limited data are available on molecular mechanisms governing Pb uptake, translocation, and detoxification in plants [11]. Some molecular actors in Pb homeostatic processes have been identified including some transporters (*NtCBP4, AtCNGC1, AtATM3, AtPDR12, AtPDR8* …) [12,13,14,15,16,17] and chelators (MTs, PCs, and GSH) [18]. Using heterologous microarray technology, several specific genes were identified as expressed under Pb exposure in *H. incana* [19]. Among these genes, *HiMT2b*, a MTs gene, was involved directly in Pb accumulation in aerial parts. Several genes implicated in ABA biosynthesis were reported to be overexpressed in roots and shoots of *H. incana* [19]. Moreover, research on *H. incana* is limited, and molecular mechanisms behind its Pb tolerance and accumulation are unclear due to the lack of knowledge on the genome of this plant. De novo assembly of transcriptome makes the study of RNAs feasible, even without a reference genome, by providing a large search transcriptome, characterizing and identifying genes that are expressed in roots and aboveground parts [20]. In this study, we first realized de novo assembly of RNA-seq transcriptome and gene annotation of *Hirschfeldia incana* L. transcriptome for the first time. We aimed to (i) explore the transcriptome of *H. incana* L. and (ii) find and characterize transcriptional changes that occur in this species in response to Pb stress.

## 2. Materials and Methods

### 2.1. Plant Material

**Figure 1:**
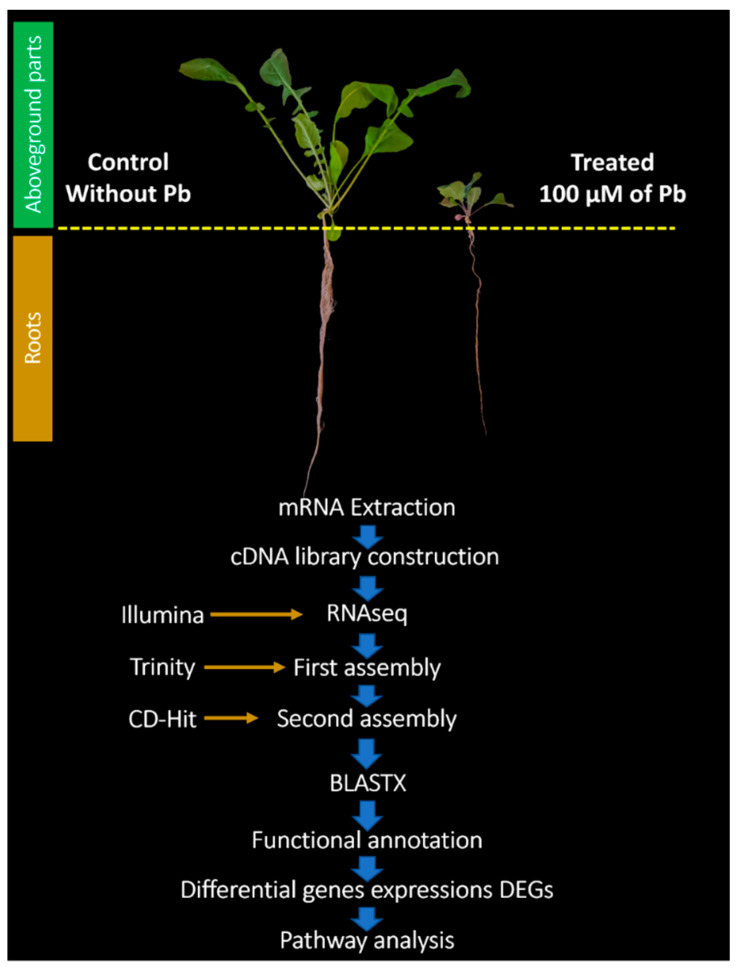
Experimental design for RNAseq and pipeline.

Seeds of *Hirschfeldia incana* were collected from Oued El Heimer Pb mining site (34°26′88″ N–1°54′03″ W) which is located in the south of Oujda city in the eastern region of Morocco [21]. Pb highly contaminates the soil of this area with concentrations higher than 18,626 mg kg^−1^ DW [8,11,22]. Two-week-old *H. incana* seedlings were cultivated in hydroponic Broughton and Dilworth (BD) medium [22] for 15 days. After acclimation, *H. incana* plants were transferred to BD medium (without phosphate) and added with 100 μM of Pb(NO_3_)_2_. This concentration used was based on the physiological and molecular responses of *H. incana* under Pb stress [8,10,20]. Control plants were cultivated in free phosphate BD medium without Pb. Plants (3 replicates per treatment) were harvested after 15 days of treatment for further experiments (Figure 1).

### 2.2. mRNA Extraction

mRNA was extracted from the roots and aboveground parts of *H. incana* plants. mRNA extraction was carried out by using the ReliaPrep^TM^ RNA Tissue miniprep System (Promega, USA) kit. A post-treatment with an Invitrogen DNA free^TM^ kit (Thermo Fisher Scientific, Waltham, MA, USA) eliminates DNA contamination. The concentrations of mRNA were assayed using Nanodrop 2000 (Thermo Scientific, Waltham, MA, USA). The quality of mRNA samples was assayed using an Agilent 2100 Bioanalyzer (Agilent Technologies Canada, Inc., Mississauga, ON, Canada), and only mRNA samples with RIN ≥7 were used for further analyses (Figure 1).

### 2.3. cDNA Construction and Sequencing

In total, 12 mRNA samples were submitted to the Genomic platform INRAE center of Toulouse Occitanie, France, for further analyses, namely mRNA quality checking, cDNA preparation, and PE mRNA sequencing. The cDNA library was constructed and then sequenced using Illumina Inc to obtain a length of approximately 150 bp (Figure 1).

### 2.4. De Novo Assembly of Transcriptome

Quality control of the sequences generated by Illumina was assayed using the Fastqc v0.11.9 tool. Reads were used for de novo assembly using Trinity tool v2.14.0, with the default parameters such as K-mers equal to 25 and a minimum length equal to 200 bp [23]. After that, a second assembly was carried out using CD-HIT (http://weizhong-cluster.ucsd.edu/cd-hit/with 95% identity) (accessed on 10 June 2022) [24]. The quality of the secondary assembly was then evaluated using BUSCO tool version 5.4.3 [25]. In addition, the reads were mapped again to the transcripts generated by CD-Hit using the Bowtie2 tool v2.4.5 (http://bowtie-bio.sourceforge.net/bowtie2/index.shtml) (accessed on 10 June 2022) (Figure 1). The transcripts generated in the study are available online: http://genoweb.toulouse.inra.fr/~mzouine/Hi_Incana/ (accessed on 10 June 2022).

### 2.5. Annotation and Function Profiling

Annotation of assembled transcripts was performed with BLASTX (E-Value 1e^−10^) on a local server using protein sequences of *Arabidopsis thaliana* downloaded from the ensemblgenomes.org database (http://ftp.ensemblgenomes.org/pub/plants/release-52/fasta/arabidopsis_thaliana/pep/Arabidopsis_thaliana.TAIR10.pep.all.fa.gz) (accessed on 10 June 2022). The BLASTX results (file represents each transcript ID with their TAIR ID) were combined with the Gene Ontology (GO) annotation file of *Arabidopsis thaliana* (https://www.arabidopsis.org/download_files/GO_and_PO_Annotations/Gene_Ontology_Annotations/gene_association.tair.gz) (accessed on 10 June 2022) using Excel software 2021 for retrieving GO terms for each transcript (Figure 1). With these GO terms, all transcripts identified are classified into biological process (BP), molecular function (MF), and cellular component (CC) domains. Then, the ShinyGO v0.76.1 tool (http://bioinformatics.sdstate.edu/go/) (accessed on 10 June 2022) [26] and REVIGO tool (http://revigo.irb.hr/) (accessed on 10 June 2022) [27] were used to classify GO terms and to graphically represent the transcript functions.

### 2.6. Transcript Estimation and DEG Analysis

For the quantification of gene expression, we mapped the reads of each replicate against the transcriptome generated by CD-HIT using the galaxy server (https://usegalaxy.org) (accessed on 10 June 2022). In this analysis, the RSEM v1.3.3 algorithm [23] was used for transcripts estimation, then bowtie2 v2.4.5 (http://bowtie-bio.sourceforge.net/bowtie2/index.shtml) (accessed on 10 June 2022) was used as a tool for mapping the reads onto the transcriptome.

DEGs were inferred based on the normalized counts with R software by using the DESeq2 v3.15 package (https://bioconductor.org/packages/release/bioc/html/DESeq2.html) (accessed on 10 June 2022), and results were extracted using the restrictive method “Bonferroni” with a *p*-adj value <0.05 [28]. After that, the Venn-Diagram v1.7.3 algorithm was used to determine the numbers of DEGs shared between control and Pb-treated plants in roots and aboveground parts [29]. Then, the Gene Ontology and Enrichment Analysis tool available online (http://bioinformatics.sdstate.edu/go) (accessed on 10 June 2022) and REVIGO tool [27] were used for functional annotation of expressed genes identified in roots and aboveground parts and to determine overrepresented GO terms across BP, MF, and MF domains. A *p*-value < 0.05 was used. The homologous genes found using BLASTX were used for pathway annotation through the KEGG database v103.1 (*Arabidopsis thaliana* used as a reference) (Figure 1). Roots and aboveground parts were harvested from six-week-old plants cultivated on Pb at 100 µM and the control. mRNAs were extracted (three replicates per condition) and sequenced using Illumina. Workflow of transcriptome analyses was performed. Whole transcriptome data were obtained and assembled. The assembled transcriptome was directly mapped onto the *A. thaliana* reference genome and then annotated and DEGs were measured to identify up- and downregulated genes under Pb exposure.

## 3. Results and Discussion

### 3.1. mRNA-seq and De Novo Transcriptome Assembly

mRNA samples extracted from roots and aboveground parts of *Hirschfeldia incana* plants were of high-quality RNA integrity number (superior to 7). The sequencing of mRNA generated millions of sequences with a length equal to 150 bp. An evaluation of the quality of all reads was performed using Fastqc. The results indicate that all reads (about 797.83 million reads) were of high quality with a phred-score ≥30. The percentage of GC was 44–47% for all the reads (Table S1). First, assembly analysis using the Trinity tool [23] generated 216,315 (211 Mb) transcripts with an average length equal to 979.57 bp and an N50 = 1304 bp. The initial transcriptome assembled reported about 44.47 percent of GC content. The second transcriptome assembly using the CD-HIT tool [24], with 95 percent of identity, produced 77,491 (77 Mb) transcripts (Table 1). The number of transcripts found in this study was higher than that obtained in the transcriptome assembly in *Brassica juncea* with 53,669 transcripts [30] and lower than that mentioned in the reported transcriptome assembly in *Brassica napus* with 161,537 transcripts [31] (Table 1). The secondary transcripts of *Hirschfeldia incana* were characterized by N50 of 1330 bp and an average length of 959.83 bp. These findings were higher than those obtained in two species of the Brassicaceae family, namely *Brassica napus* (N50 = 1093 pb, average length = 693 pb) [31] and *Brassica juncea* (N50 = 1282 pb, average length = 953 pb) [30] but lower than those of *Brassica nigra* (N50 = 1482 pb, average length = 1173 pb) [32]. The secondary assembly of the *H. incana* transcriptome revealed 43.83% of GC content (Table 1). This percentage was slightly lower than GC content reported in *B. juncea* (44.38%) [30], and higher than GC content reported in *Brassica nigra* (37%) [32]. The reads were mapped again to the secondary assembly using the bowtie2 aligner tool to evaluate the consistency of the transcriptomes generated by CD-Hit. All reads were successfully mapped to the secondary assembly with a percentage higher than 93.5 percent (Table S1). The quality of the transcriptome assembly was verified using the BUSCO score algorithm. As shown in Table 1, a high percentage of BUSCO score was obtained; about 94.2% were complete, 2.8% were fragmented, and 3.0% were missing (Table S1). The percentage of the BUSCO reported in the present study was slightly lower than that reported in *Brassica nigra* [32].

**Figure 2:**
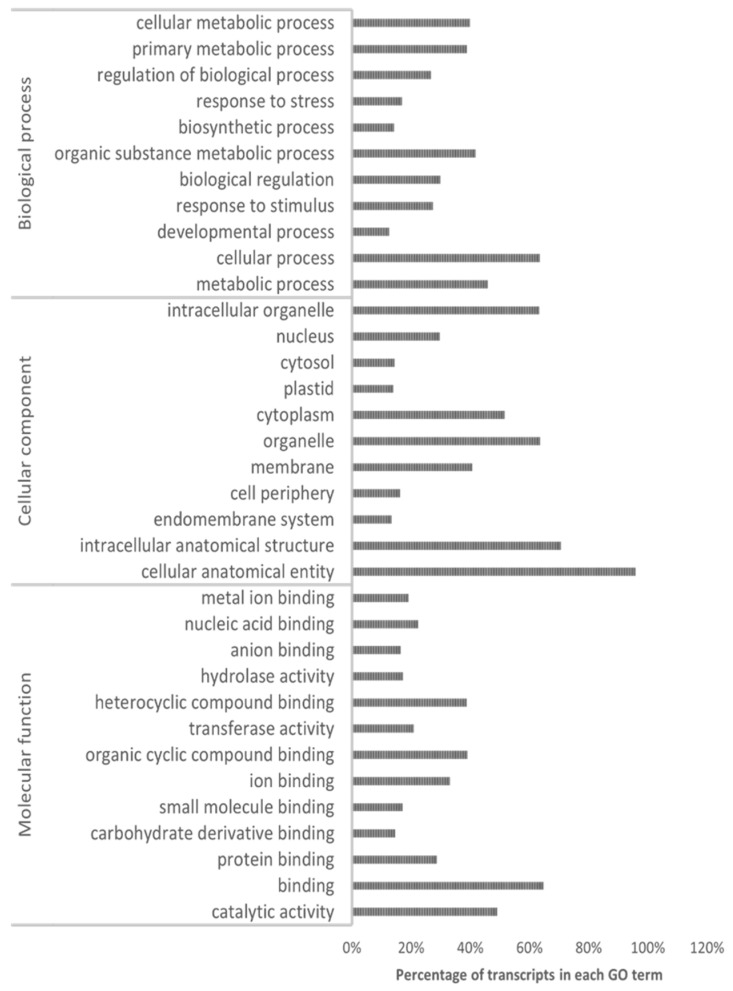
The GO classification of *H. incana* transcripts in response to Pb stress.

The annotation of 77,491 secondary transcripts was performed using BLASTX on the protein database of *Arabidopsis thaliana*. About 72,306 annotated transcripts were obtained, with 11,862 duplicate transcripts removed and 60,444 unique transcripts (Table S3). GO annotation of *H. incana* transcripts was performed using the gene ontology annotation file of *Arabidopsis thaliana*. Afterward, GO annotations were classified into three essential domains, BP, CC, and MF (Figure 2; Table S3). Out of 60,444 annotated transcripts, 60,395 transcripts possess GO. About 13,046 own unique GO terms. In total, 7048 transcripts belong to the BP domain, with 1170 BP functions highly overrepresented (*p* < 0.05) (Table S3). The majority of them belong to the cellular process “GO:0009987”, metabolic processes “GO:0008152”, response to stimulus “GO:0050896”, and response to stress “GO:0006950” (Figure 2; Table S3). Similar findings were reported in *Brassica napus* [31]. For MF, a total of 4535 terms were found (Figure 2; Table S3). The most overrepresented activities were binding “GO:0005488” followed by catalytic activity “GO:0003824” (Figure 2; Table S3). The same activities were shown most represented in the MF category of *Brassica napus* [31]. However, 1463 GOs were found in the CC, most of them located in organelle “GO:0043226”, cytoplasm “GO:0005737”, and membrane “GO:0016020” (Figure 2; Table S3). Similar results were reported in *Brassica juncea* [30]. In addition, pathway analyses were performed using the KEGG database (*Arabidopsis thaliana* as the reference) to identify the pathways and understand the possible gene interactions. In total, 13,046 transcripts were assigned to 138 pathways. Among these pathways, metabolic pathways, biosynthesis of secondary metabolites, and plant hormone signal transduction were the top nine pathways represented by transcripts (Table S3). Our results indicate that the transcriptome generated in this study is successfully assembled and characterized by high quality, with relatively complete annotation information. The transcriptome assembly obtained provides helpful reference data and can be used to conduct subsequent functional genomics research in *Hirschfeldia incana*.

### 3.2. Differential Expressed Genes (DEGs) under Pb Exposure

**Figure 3:**
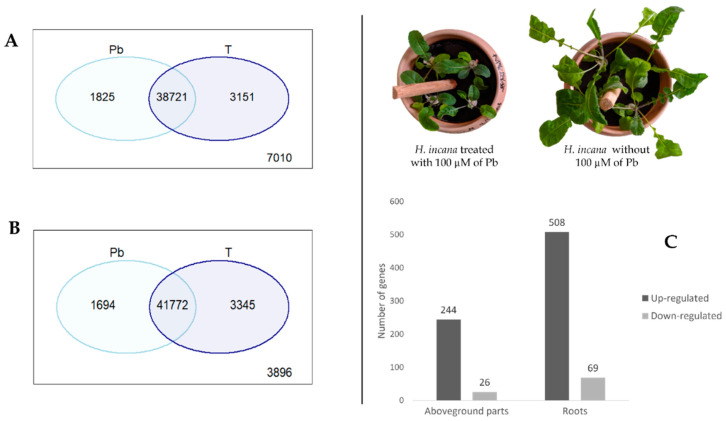
Venn diagram and DEGs in roots and aboveground parts of *H. incana*: (**A**) Venn diagram for control (T) and treated (Pb) in aboveground parts; (**B**) Venn diagram for control (T) and treated (Pb) in roots; and (**C**) number of DEGs in different tissues.

For *H. incana*, the Venn diagram results indicate that most transcripts were shared between treated (Pb) and control (T) conditions. About 38,721 and 41,772 transcripts were shared between the two conditions in aboveground parts and roots, respectively (Figure 3A,B). Using the empirical criterion of ≥2-fold change for upregulated and ≤−2-fold change for downregulated and restrictive method “Bonferroni” with *p*-value < 0.05, a total of 577 and 270 genes were differentially expressed (DE) under Pb exposure in roots and aboveground parts, respectively (Figure 3C, Table S5). For roots, among 577 DEGs, 508 transcripts were upregulated, whereas 69 were downregulated under Pb stress (Figure 3C, Table S5). Without using the restrictive method, several studies detected a high number of genes in response to heavy-metal stress. For example, a total of 4614 DEGs were detected in roots of *Raphanus sativus* L., with 2154 upregulated genes and 2460 downregulated genes under Pb stress [33]. Another study reported 4682 genes expressed and 3599 inhibited in *B. juncea* root under Cd stress [34]. In our study, among the 270 genes, 244 were over-expressed, whereas 26 genes were inhibited in aboveground parts of *H. incana* in response to Pb stress (Figure 3C, Table S4). Other authors reported that under Pb stress, about 1641 over-expressed and 2884 inhibited genes in shoots of Pb hyper-tolerant species, namely *Fagopyrum tataricum* [3]. A study on *B. juncea* revealed 2021 upregulated and 992 downregulated genes in the shoots in response to Cd stress [34].

**Figure 4:**
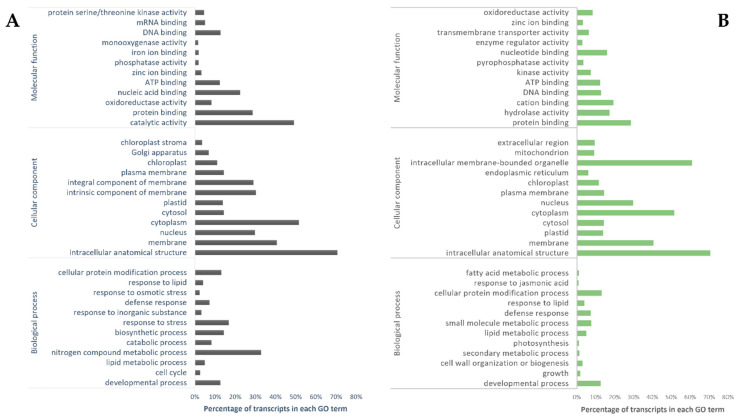
Top 12 GO classification of upregulated genes in roots (**A**) and aboveground parts (**B**) of *H. incana* under Pb exposure.

The GO enrichment analysis was performed and DEGs were annotated and classified into BP, CC, and MF domains (Table S5). For *H. incana* roots, the GO enrichment analysis of upregulated genes identified 432 CC, 221 MF, and 96 CC terms (Figure 4A, Table S5). Response to stress “GO:0006950”, biosynthetic process “GO:0009058”, and developmental process “GO:0032502” are the most represented GO terms in the BP domain (Figure 4A).

For the MF domain, catalytic activity “GO:0003824” and protein binding “GO:0005515” were the most represented (Figure 4A). In the CC domain, cytoplasm “GO:0005737”, membrane “GO:0016020”, plastid “GO:0009536”, and cell wall “GO:0009505” were overrepresented. Under heavy-metal stress, including Pb, the cell wall is the first barrier that protects the cell against Pb entry into the cytoplasm [35]. The cell wall contains several component such as polysaccharides and proteins that may bind Pb ions [36]. Such binding severely limits the Pb transport into the cell, thereby allowing cellular metabolism maintenance [37]. Our results showed that the CC group of genes is particularly enriched by Pb stress in roots of *H. incana* and contains genes expressed in the cell walls and cell membranes. Additionally, genes associated with the biosynthesis of polysaccharides such as cellulose were shown to be significantly upregulated (LogFC = 3.31) in response to Pb stress, which may result in considerable cell wall thickening [3].

Transcripts identified in *H. incana* roots exposed to Pb were related to the catalytic activity, protein binding, and nitrogen compound metabolic process. Similarly, in *R. sativus* L. roots, different BP and MF were reported under Pb stress [33]. These overrepresented GO terms were also related to catalytic activity and explained by the adverse effects of heavy metals in plants resulting in the inhibition of biomass production and plant growth [38].

For downregulated transcripts, the most represented GO, identified in roots of *H. incana* in response to Pb stress, are related to carbohydrate metabolic process “GO:0005975”, defense response “GO:0006952”, and proteolysis “GO:0006508”, in BP (Table S5). Protein binding and DNA binding are most represented in the MF domain (Table S4). For CC, the overrepresented downregulated transcripts were located in the intracellular membrane-bounded organelle, cytoplasm, and nucleus (Table S5). In *R. sativus* roots, cell-wall organization and catalytic activity were the most represented in BP and MF domains, respectively, in downregulated genes under Pb stress [33].

**Figure 5:**
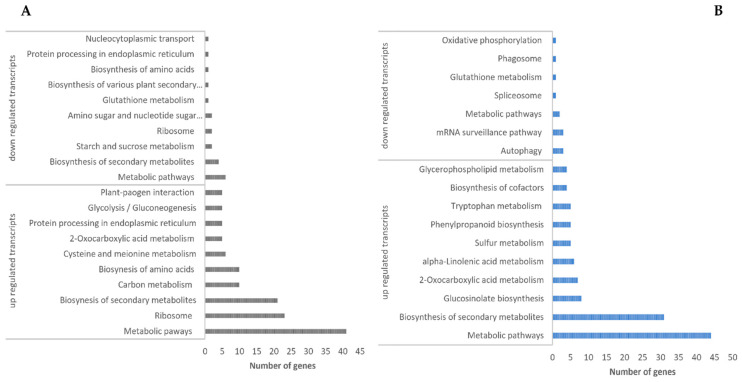
Top pathways of DEGs in roots (**A**) and aboveground parts (**B**) of *H. incana* in response to Pb exposure.

The KEGG pathway enrichment analysis of upregulated transcripts in *H. incana* L. roots identified 67 enriched pathways in response to Pb stress (Table S5), 10 of which were overrepresented (Figure 5A, Table S5). Metabolic, biosynthesis of secondary metabolites, and carbon metabolism were the most significantly enriched pathways responding to Pb stress. For downregulated transcripts, 17 pathways were reduced in response to Pb stress (Figure 5A, Table S5), suggesting that pathways could differ between species in response to Pb stress [33].

In the aboveground parts, GO enrichment analysis of upregulated genes identified 382 BP terms, 245 MF terms, and 64 CC (Figure 4B, Table S5). For the upregulated transcripts, developmental process “GO:0032502”, defense response “GO:0006952”, and small molecule metabolic process “GO: 0044281” were the most represented in the BP domain (Figure 4B). In the MF domain, protein binding, cation binding, hydrolase activity, and oxidoreductase activity and those related to the CC domain include intracellular anatomical structure, cytoplasm “GO:0005737”, membrane “GO:0016020”, cell wall, chloroplast “GO:0009507”, and Golgi apparatus GO:0005794 (Figure 4B; Table S5) were the most represented. These activities and functions were reported in DEGs in *F. tataricum* shoots in response to Pb exposure [3]. For downregulated genes detected in *H. incana* aboveground parts in response to Pb stress, 42 GO overrepresented in the BP domain include root morphogenesis “GO:0010015”, cellular protein localization “GO:0034613”, and response to light stimulus “GO:0009416” (Table S5). For MF, 25 GO including protein binding “GO:0005515”, and nucleic acid binding “GO:0003676” (Table S5), and 24 GO related to CC include cytoplasm “GO:0005737”, and nucleus “GO:0005634” (Table S5). However, 100% of the 10 most up- and down-expressed transcripts in roots and aboveground parts are associated with a GO term (Table 2). The KEGG analysis showed 57 different pathways affected by Pb stress in aboveground parts. The DEGs analysis revealed that the most upregulated transcripts were associated with different pathways, including metabolic, glucosinolate biosynthesis, and alpha-linolenic acid (Figure 5B; Table S5). Different pathways are identified in shoots of *F. tataricum* under Pb exposure [3]. For the downregulated genes, many pathways, such as plant autophagy and metabolic are overrepresented in aboveground parts of *H. incana* L. under Pb stress (Figure 5B; Table S5).

Previous studies on *H. incana* response to Pb reported that this perennial plant tolerates and accumulates a high level of Pb in its aboveground parts [8,10,20]. These results suggest that *H. incana* has developed adaptative mechanisms to enhance the capacity to survive, tolerate, and accumulate Pb and are certainly implicated in the activation of many essential transporters. A multitude of transporter families, including *OCT*, *CNGC*, and *ABC* which have been characterized as playing a crucial role in heavy-metal uptake, transport, distribution, and plant tolerance [39], were identified as upregulated in the present study. γ-aminobutyric acid (*GABA*) transporter was upregulated in *H. incana* aboveground parts in response to Pb stress. *GABA* is implicated in several physiological activities and contributes to the tolerance of plants to different stresses, including heavy metals [40]. Previous studies showed that *GABA* was upregulated during Zn and Cr stress in *Nicotiana tabaccum* L. [41] and *Brassica juncea* [42]. *AVP1*, a proton pump located in the vacuolar membrane that enhances cadmium tolerance and accumulation in tobacco plants [43], was shown upregulated in aboveground parts of *H. incana* under Pb stress (Table S5). The *ATP-Mg/Pi* transporter (*APC3*: mitochondrial substrate carrier family protein) was shown upregulated in aboveground parts of *H. incana* during Pb stress (Table S5). *AtAPC1* and *AtAPC3* are known to transport phosphate compounds such as AMP, ADP, and ATP [44]. Abiotic stress, such as heavy-metal stress, causes increased energy demand [45], which requires high consumption of ATP and ADP [45]. The high energy consumption may be related to the expression of the *APC3* gene under Pb stress.

Several studies reported that heavy metals affect the absorption of Mg and the photosynthesis process. For example, Cr reduces the absorption of Mg resulting in lower chlorophyll content [46]. Pb causes an increase in the activity of some enzymes, specifically chlorophyllase. Additionally, Pb could modify and change the activity of photosynthesis [47]. In the present study, the treatment with 100 µM of Pb decreases the expression level of the Mg ion transporter and can directly reduce the photosynthesis activity under Pb stress. In this study, several magnesium ion transporters of the *MRS2* family were shown regulated by Pb. *MRS2-3, MRS2-7*, and *AtMRS2-11,* essential in transporting Mg in the chloroplast membrane system [48], were shown downregulated in aboveground parts of *H. incana* under Pb exposure

In *H. incana* roots, many transporters are significantly upregulated in response to Pb, such as *CNGC5*. *CNGC* family transporters are channels whereby metal ions can enter the cells and are involved in tolerance to heavy-metal stress [49]. In *Arabidopsis thaliana*, many members of the *CNGC* family such as *AtCNGC11* and *AtCNGC15* have been recorded in plant tolerance and uptake of Pb^2+^ and Cd^2+^ [50]. In the current study, the *HAK5* gene involved in K^+^ uptake [51] is shown upregulated in the root of *H. incana* in response to Pb stress. Metal toxicity induces oxidative stress, leading to ROS production and modification of several physiological activities, including degradation of several enzymes, amino acids, and proteins [52]. Potassium activates the antioxidant defense in plants and therefore increases oxidative stress tolerance by minimizing NADPH’s activity and controlling photosynthetic activity, which helps to limit ROS production [53]. In the present study, upregulation of the *HAK5* gene in *H. incana* roots suggests the implication of potassium transporters in the reduction of the damage caused by Pb. To sum up, several genes coding for *ABC*, *OCT*, *MRS,* and *CNGC* transporter families were upregulated and may play an essential role in *H. incana* Pb tolerance. Further functional analyses are necessary to validate their specific implication in response to Pb exposure.

Several genes involved in regulation, biosynthesis, and transport of glucosinolates such as *SOT18* (sulfotransferase), *GTR1* (major facilitator superfamily protein), *APK2* (adenylyl-sulfate kinase 2), *JAL23*, *BASS5*, *BGLU32* (beta-glucosidase 32), and *IGMT5* in aboveground parts and *SDI1* (sulphur deficiency-induced 1) in roots were upregulated in *H. incana* in response to Pb stress. Our finding is in accordance with several reports mentioning that under Zn, Cd, and Pb the biosynthesis of glucosinolates was higher in other *Brassica* species [54,55]. These authors suggest that glucosinolates play an important role in heavy-metal tolerance in different *Brassica* plant species [54,55].

Under Pb exposure, several transcription factors (TFs) were shown upregulated in aboveground parts such as *WRKY40*, *MYB95,* and *MYB15* and in roots of *H. incana* such as *ERF021* and *MYB108*. Transcription factors are implicated in the control of different transcriptional activities under heavy-metal stress [56,57]. Several studies reported an over-expression of *MYB* and *WARKY* families in different plants species in response to heavy-metal exposure [57,58]. *MYB4* plays an essential role in controlling gene expression in different pathways, specifically the phenylpropanoid pathway. In the present study, C*4H* (cinnamate-4-hydroxylase) and *CCR2* (Cinnamoyl-CoA reductase 2), implicated in phenylpropanoid biosynthetic activities, were upregulated in aboveground parts and roots of *H. incana*, respectively, under Pb exposure. Some authors reported that the phenylpropanoid pathway is activated under heavy-metal exposure and involved in the protection of plants against ROS [59]. This type of TF is implicated directly in the control of glucosinolate production that is increased in *N. caerulescens* shoots after exposure to Cd [60]. In addition, several genes implicated in jasmonates (JAs) biosynthesis are shown to be significantly upregulated in aboveground parts of *H. incana* in response to Pb stress. Various TFs increase the concentrations of JAs and jasmonyl isoleucine by controlling the activation of some essential genes implicated directly in JAs biosynthesis such as *LOX*, *AOS*, and *AOC* in response to heavy-metal stress [61]. JAs are also essential for the induction of *ERF1* in response to HMs stresses [62]. *ERFs* were reported as playing an essential role in the response of plants to Cd and Pb stress by regulating several genes’ expression [33].

### 3.3. Jasmonates (JAs) and Plant Response to Pb—A Case Study

**Figure 6:**
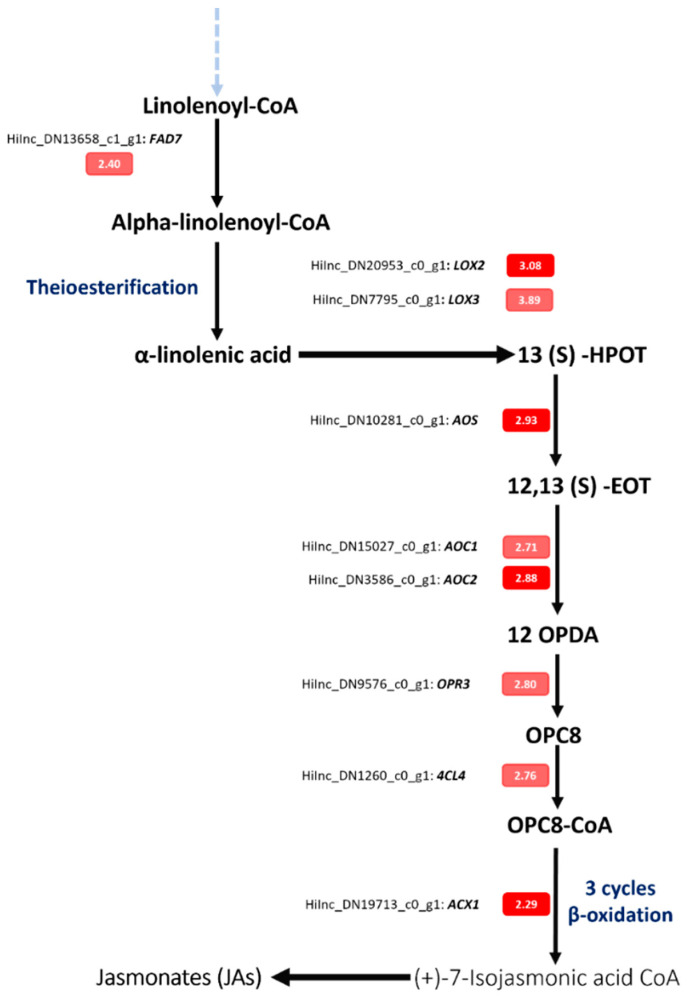
Expression level of genes implicated in the JAs biosynthesis pathways in *H. incana* under Pb exposure. The red color represents the genes selected using the restrictive method “Bonferroni”, and the light red represent the genes selected without using the restrictive method.

Jasmonates (JAs) are signaling molecules involved in plant development and plant responses to biotic and abiotic stresses [63]. In the present study, whole transcriptomic analysis of *Hirschfeldia incana* showed that under Pb stress several upregulated genes in roots and aboveground parts are involved in JAs biosynthesis (Figure 6). Seven genes were characterized as DEGs in aboveground parts, namely *LOX2*, in chloroplast, *AOS* in thylakoid and plastid, *AOC2* and *AOC4* in plastid, *ACX1* and *KAT5* in peroxisome, and the *JAZ10* gene located in the nucleus (Table S4). In *H. incana* roots, the *JAZ10* gene was shown upregulated under Pb exposure. Several studies reported that JAs synthesis and pathways are affected by HM stress. For example, Cd activates the expression of genes involved in JAs synthesis, thus, increasing the concentrations of JAs in the root tissues of *Arabidopsis thaliana* [64]. The authors mentioned that *AOS* and *AOC* genes were upregulated in *Arabidopsis* under Pb exposure [65]. A study by [66] reported that JAs are synthetized through several steps from lipoxygenases, followed by oxygenation of α-linolenic acid. According to [67,68], the JA biosynthesis is controlled by two crucial reduction and oxidation steps (Figure 6). In this study, DEGs analysis reveals that the JAs pathway is activated by Pb stress. This finding suggests that JAs play an essential role in *H. incana* response to Pb stress.

Alpha-linolenic acid is an essential element for the JA biosynthesis [66]. In our study, the *FAD7* (fatty acid desaturase 7) gene was significantly upregulated with logFC = 2.40 in response to Pb stress (Table S5; Figure 6). The *FAD* gene is implicated directly in the biosynthesis of unsaturated fatty acid such as α-linolenic acid content and adversely affects JAs in response to heavy-metal stress [69]. Previous studies mentioned that fatty acid desaturase genes are essential for stabilizing the plant membrane under abiotic stress, including heavy metals, by producing high amounts of fatty acids [70,71]. Fatty acids are essential components of cellular membranes and can play an important role in structural barriers for minimizing the effects of Pb in plants [72]. They contribute to inducible stress tolerance through controlling membrane fluidity [73]. Alpha-linoleic acid can also be used as a substrate for JA biosynthesis. Alpha-linolenic acid and JAs then emerge as a strategy to withstand Pb toxicity in *H. incana*.

## 4. Conclusions

This work presents the first evidence on the RNA-seq, de novo assembly, and functional annotation of the Greek mustard (*Hirschfeldia incana* L.) transcriptome, and the differential expression genes (DEGs) in roots and aboveground parts in response to Pb stress. The de novo assembly produced 77,491 transcripts, creating a reference transcriptome with an N50 of 1330 bp and 43.83 percent of GC. We report more than 60,444 annotated transcripts in *H. incana*. In total, 577 and 270 DEGs were identified in response to Pb stress in *H*. *incana* roots and aboveground parts, respectively. The GO term enrichment revealed that in *H. incana,* most identified genes involved in biosynthetic processes were significantly upregulated under Pb stress. Whereas genes involved in carbohydrate metabolic process, and defense response were significantly downregulated in roots. In aboveground parts, the majority of genes significantly upregulated are involved in lipid metabolic process, defense response, and response to jasmonic acid. In addition, several genes implicated in jasmonates biosynthesis were significantly upregulated under Pb exposure, suggesting that *H. incana* uses the JAs pathway as a strategy to alleviate Pb stress. The dataset generated in this study also contributes to the molecular resources of *Hirschfeldia incana*.

## Figures and Tables

**Table 1:** Transcriptome comparison of four Brassicaceae species including *H. incana, B. napus* [31], *B. juncea* [30], and *B. nigra* [32].

Initial Transcripts (TRINITY)	*H. incana*	*B. napus*	*B. nigra*	*B. juncea*
Total transcripts	216,315	-	-	-
Total assembled bases	211,894,927	-	-	-
Average length (pb)	979.57	-	-	-
N50	1304	-	-	-
Percent GC	44.47	-	-	-
**Secondary transcripts (CD-HIT)**				
Total transcripts	77,491	161,537	NA	53,669
Total assembled bases	74,378,239	111,953,629	NA	51,151,545
Average length (pb)	959.83	693	1173	953
N50	1330	1093	1482	1282
Percent GC	43.83	NA	37	44.38
BUSCO %	94.2%	NA	97%	NA
**Number of genes**	**50,707**	**NA**	**56,331**	**NA**

**Table 2:** Example of the most upregulated DEGs (A) and downregulated DEGs (B) in roots and aboveground parts of *H. incana* in response to Pb stress. Bold numbers indicate the standard error for the Log foldchange.

**(A)**				
**Transcript ID**	**Transcript Name**	**Log2FC**	***p*** **-Value**	**N° of GO Terms**
**Up-Regulated Transcripts in aboveground Parts of *H. incana***				
**HiInc_DN6940_c0_g2**	Noc2p family	10.56 **1.94**	5.43 × 10^−8^	8
**HiInc_DN64666_c0_g1**	RNA-directed DNA polymerase (Reverse transcriptase)-related family protein	8.55 **1.47**	6.97 × 10^−9^	3
**HiInc_DN54630_c0_g1**	Translation initiation factor IF3-4	7.31 **1.40**	2.02 × 10^−7^	8
**HiInc_DN38797_c0_g1**	Peroxidase	6.07 **1.84**	1.57 × 10^−11^	4
**HiInc_DN8233_c0_g1**	Inorganic pyrophosphatase 2	5.65 **0.86**	6.74 × 10^−11^	6
**HiInc_DN250_c0_g1**	Syringolide-induced protein	5.28 **0.88**	2.89 × 10^−9^	7
**HiInc_DN35271_c0_g1**	Beta-glucosidase 32	5.13 **1.64**	3.11 × 10^−12^	6
**HiInc_DN5802_c0_g2**	Monogalactosyldiacylglycerol synthase 2	5.10 **0.57**	4.03 × 10^−19^	9
**HiInc_DN12789_c0_g1**	REF/SRPP-like protein	5.02 **0.69**	3.25 × 10^−13^	7
**HiInc_DN1096_c0_g4**	Salicylate/benzoate carboxyl methyltransferase	4.89 **0.92**	1.36 × 10^−7^	5
**Up-regulated transcripts in roots of *H. incana***				
**HiInc_DN6451_c0_g2**	Endochitinase	11.68 **1.75**	2.60 × 10^−11^	7
**HiInc_DN64488_c0_g1**	ATPase 9	10.29 **1.96**	1.52 × 10^−7^	4
**HiInc_DN5848_c0_g1**	Accelerated cell death 11	9.89 **1.96**	5.01 × 10^−7^	7
**HiInc_DN27683_c0_g1**	Putative endonuclease or glycosyl hydrolase	9.74 **1.32**	2.38 × 10^−13^	2
**HiInc_DN6940_c0_g1**	Noc2p family	9.47 **1.49**	2.56 × 10^−10^	4
**HiInc_DN10792_c0_g1**	Nucleic acid-binding	8.82 **1.48**	3.06 × 10^−9^	3
**HiInc_DN62699_c2_g1**	BED zinc finger and hAT dimerization domain-containing protein DAYSLEEPER	8.70 **1.63**	1.03 × 10^−7^	1
**HiInc_DN63847_c0_g1**	Alpha carbonic anhydrase 2	8.36 **1.29**	1.20 × 10^−10^	4
**HiInc_DN13103_c0_g1**	60S ribosomal protein L10a-2	7.81 **1.33**	5.24 × 10^−9^	2
**HiInc_DN8575_c1_g1**	Disease resistance protein (TIR-NBS class)	7.77 **1.39**	2.71 × 10^−8^	13
**(B)**				
**Transcript ID**	**Transcript name**	**Log2FC**	***p*** **-value**	**N° of GO terms**
**Down-regulated transcripts in aboveground parts of *H. incana***				
**HiInc_DN38456_c0_g1**	transmembrane protein, putative (DGR2)	−2.10 **0.25**	1.20 × 10^−16^	1
**HiInc_DN19265_c0_g1**	Chaperone DnaJ-domain superfamily protein	−2.17 **0.43**	7.06 × 10^−7^	5
**HiInc_DN12488_c0_g1**	Thioredoxin-like protein CXXS1	−2.19 **0.34**	3.79 × 10^−6^	2
**HiInc_DN54188_c0_g1**	Tetratricopeptide repeat (TPR)-like	−2.24 **0.45**	1.02 × 10^−6^	2
**HiInc_DN9080_c0_g1**	Tubulin alpha chain	−2.29 **0.39**	4.30 × 10^−9^	15
**HiInc_DN33913_c0_g1**	Probable aquaporin PIP2-8	−2.37 **0.42**	2.20 × 10^−8^	3
**HiInc_DN7675_c0_g1**	Gamma vacuolar processing enzyme	−2.51 **0.35**	2.21 × 10^−12^	12
**HiInc_DN10024_c0_g1**	RNA-binding (RRM/RBD/RNP motifs)	−2.75 **0.55**	8.13 × 10^−7^	6
**HiInc_DN18907_c0_g1**	RmlC-like cupins superfamily protein	−2.79 **0.52**	7.56 × 10^−8^	2
**HiInc_DN20249_c0_g1**	Ribosomal protein S3Ae	−3.27 **0.53**	8.09 × 10^−10^	8
**Down-regulated transcripts in roots of *H. incana***				
**HiInc_DN1121_c0_g2**	RmlC-like cupins superfamily protein	−2.02 **0.22**	8.16 × 10^−19^	3
**HiInc_DN2301_c0_g1**	P-loop containing nucleoside triphosphate hydrolases superfamily protein	−2.06 **0.33**	5.82 × 10^−10^	4
**HiInc_DN315_c5_g1**	Receptor-like protein 30	−2.07 **0.39**	1.50 × 10^−7^	6
**HiInc_DN6047_c0_g1**	Protein kinase superfamily protein	−2.10 **0.39**	1.22 × 10^−7^	2
**HiInc_DN10269_c1_g2**	UDP-glycosyltransferase 76B1	−2.42 **0.49**	1.07 × 10^−6^	8
**HiInc_DN38838_c0_g1**	60S acidic ribosomal protein P2-3	−2.45 **0.33**	2.33 × 10^−13^	2
**HiInc_DN16964_c0_g1**	Probable LRR receptor-like serine/threonine-protein kinase	−2.48 **0.34**	8.71 × 10^−10^	2
**HiInc_DN22651_c0_g3**	Glutathione S-transferase F3	−2.51 **0.26**	7.18 × 10^−7^	5
**HiInc_DN24519_c1_g1**	Probable LRR receptor-like serine/threonine-protein kinase	−2.51 **0.50**	5.27 × 10^−7^	7
**HiInc_DN13802_c0_g1**	Tricyclene synthase	−2.64 **0.26**	5.76 × 10^−24^	8

## Data Availability

The transcripts generated in the study are available online: http://genoweb.toulouse.inra.fr/~mzouine/Hi_Incana/.

## Supplementary Materials

The following supporting information can be downloaded at: https://www.mdpi.com/article/10.3390/cimb44100318/s1, Table S1: RNA-Seq data summary; Table S2: Results of BlastX; Table S3: Annotations of total transcripts; Table S4: Gene expressions sorted by normal method; Table S5: Gene expressions sorted by restricted method; Table S6: Pathways identified using KEGG.

## Abbreviations

*ACX1*	Acyl-CoA oxidase 1;
*AOC4*	allene oxide cyclase 4;
*AOS*	allene oxide synthase;
BUSCO	Benchmarking Universal Single-Copy Orthologs;
CD-HIT	Cluster Database at High Identity with Tolerance;
DEGs	differentially gene expressions;
DE	differential expressed;
GO	Gene Ontology;
GTH	glutathione;
*JAZ10*	JASMONATE ZIM-domain protein 10;
JAs	jasmonates;
KAT5	3-keto-acyl-CoA thiolase 2;
KEGG	Kyoto Encyclopedia of Genes and Genomes;
*LOX2*	lipoxygenase;
HMs	heavy metals;
MTs	metallothioneins;
PCs	Phytochelatin;
REVIGO	reduce and visualize gene ontology;
RIN	RNA Integrity Number;
ROS	reactive oxygen species;
RSEM	RNA-Seq by Expectation-Maximization;
TFs	transcription factors;
13(S)-HPOT	(9Z,11E,15Z) -(13S)-13-Hydroperoxyoctadeca-9,11,15-trienoic acid;
12,13(S)-EOT	(9Z,15Z)-(13S)-12,13-Epoxyoctadeca-9,11,15-trienoic acid;
12-OPDA	(15Z)-12-Oxophyto-10,15-dienoic acid;
OPC8	8-[(1R,2R)-3-Oxo-2-(Z)-pent-2-enylcyclopentyl] octanoate
